# High tumor amplification burden is associated with *TP53* mutations in the pan-cancer setting

**DOI:** 10.1080/15384047.2022.2128608

**Published:** 2022-09-28

**Authors:** Rushikesh S. Joshi, Amelie Boichard, Jacob J. Adashek, Razelle Kurzrock

**Affiliations:** a† School of Medicine, University of California San Diego, La Jolla, CA, USA; b† Department of Molecular Cancer Genetics, University of Strasbourg Hospitals, Strasbourg, France; Department of Oncology, The Sidney Kimmel Comprehensive Cancer Center, The Johns Hopkins Hospital, Baltimore, MD, USA; d MCW Cancer Center and Genomic Sciences and Precision Medicine Center, Medical College of Wisconsin, WIS, USA; e Worldwide Innovative Network (WIN) for Personalized Cancer Therapy, Paris, France

**Keywords:** cancer genes, next-generation sequencing, amplifications, *TP53*

## Abstract

Next-generation sequencing data is fundamentally changing the clinical management of patients with cancer. The most frequent genomic alterations in malignancy are mutations and amplifications, with a subset of tumors having multiple amplifications – “amplificators”. We sought to understand the molecular correlates of high tumor amplification burden in a pan-cancer context. Using both national registries and a single-institution dataset, our results demonstrate that cancers with *TP53* mutations (as compared to those with wild-type *TP53*) exhibited significantly higher tumor amplification burden across all datasets. Amplifications, generally associated with overexpression, may be potentially actionable secondary consequences of *TP53* mutations.

## Background

Advances in next-generation sequencing (NGS) techniques have begun to revolutionize our fundamental understanding of disease, especially cancer. The identification of specific genomic anomalies has enabled the development of molecular and immune marker-specific drugs as treatment options for various cancers.^1–5^ For instance, some patients with high tumor mutational burden appear more responsive to immune checkpoint blockade.^1,6^

However, the underlying biology driving certain genomic alteration patterns remains to be elucidated. As an example, gene amplification refers to an increase in the number of copies of a specific gene and is a prominent manifestation of genomic instability in mammalian cells.^7,8^ Gene amplifications are often present in cancer cells and can be the cause of RNA or protein overexpression.^8,9^ The occurrence of gene amplifications in early stages of cancer and the amplification of multiple genes in some tumors may suggest an underlying genomic etiology.^10,11^ While the mechanisms behind gene amplification have not been empirically determined, they are generally understood to be the result of DNA double-stranded breaks, impaired DNA replication, or dysfunction in the DNA repair machinery.^10^ Interestingly, we have observed a group of patients, dubbed ‘amplificators’, who have large numbers of gene amplifications, with or without concomitant large numbers of deleterious mutations.

**Figure 1.**
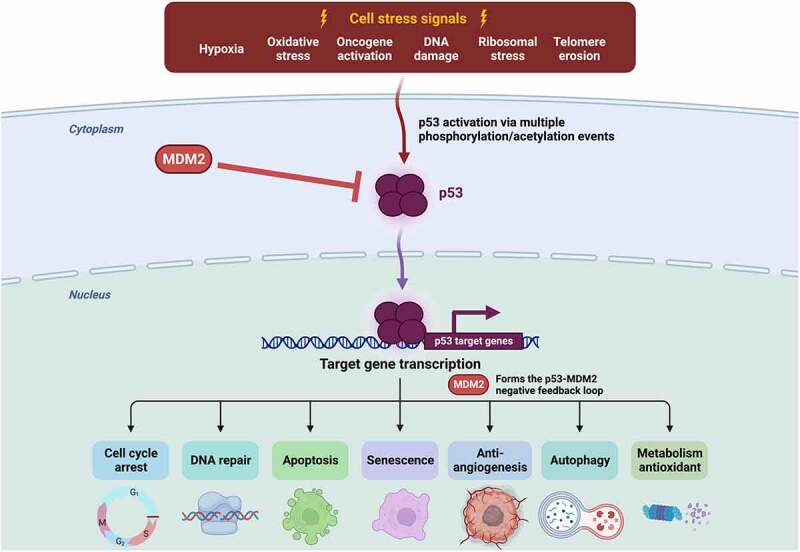
The role of *TP53* in maintaining the integrity of the genome.

In this study, we reviewed the medical records of 1,891 patients seen at the University of California, San Diego (UCSD) Moores Center for Personalized Cancer Therapy, and additionally explored 7,246 tumor samples from The Cancer Genome Atlas (TCGA). We show an association between *TP53* mutations and a high number of oncogenic gene amplifications. *TP53* is a tumor suppressor, designated the “guardian of the genome” because of its crucial role in maintaining genomic integrity (Figure 1).^11–16^ Although *TP53* alterations are considered difficult to drug, their secondary effects, such as amplifications, might be important in that the resultant overexpression levels may be actionable.

## Results and discussion

Exploratory analysis was performed to identify patients deemed to have higher proportions of gene amplifications. To define the “amplificator” phenotype, we examined samples that expressed the top 10% of tumor amplification burden, across all samples, and based on two different sequencing panels. The two panels used for sequencing were whole genome sequencing (WGS) and a panel of 315 common oncogenes from the FoundationOne CDx gene panel by Foundation Medicine (FM) (https://www.foundationmedicine.com). A total of 7,246 patient samples were included from TCGA and 1,891 patients treated at Moores Cancer Center at UCSD and sequenced by FM.

In Table 1, the differences between amplificators and non-amplificators, as determined by both WGS and the FM gene panel were tabulated for the TCGA cohort, while the UCSD cohort amplificator phenotype was determined solely by the genes in the FM panel. Within the TCGA cohort, amplificators, as defined by both WGS and the FM panel, exhibited a higher proportion of *TP53* mutated samples (38% in WGS non-amplificators vs. 62% in WGS amplificators; 37% in FM panel non-amplificators vs. 66% in FM panel amplificators; all p < .001). Additionally, the average number of WGS and FM-panel amplifications was dramatically higher in the amplificator phenotypes as defined by both criteria (1,720 WGS amplifications and 22.4 FM-panel amplifications in WGS amplificators vs. 190 WGS amplifications and 3.2 FM-panel amplifications in WGS non-amplificators; 1,562 WGS amplifications and 24.0 FM-panel amplifications in FM-panel amplificators vs. 207 WGS amplifications and 3.0 FM-panel amplifications in FM-panel non-amplificators; all p < .001). In the UCSD cohort, amplificators as defined by the FM panel similarly exhibited a higher proportion of *TP53* mutated samples (41% in FM panel non-amplificators vs. 63% in FM panel amplificators, p < .001) as well as a higher average number of FM panel amplifications per sample (14.2 FM panel amplifications vs. 1.2 FM panel amplifications). Subsequently, a secondary analysis exploring differences in gene amplifications between *TP53* mutated and *TP53* wild-type samples in both the TCGA and UCSD cohorts was then conducted (Table 1). In the TCGA cohort, when stratified by *TP53* genotype (N = 2897 *TP53* mutated vs. N = 4349 *TP53* wild-type), the *TP53* mutated patients exhibited a higher average number of amplifications in both the WGS and FM panel when compared to wild-type patients (516 WGS amplifications vs. 229 WGS amplifications and 7.7 FM panel amplifications vs. 3.4 FM panel amplifications; all p < .001). In the UCSD patient cohort (N = 824 *TP53* mutated vs. N = 1067 *TP53* wild-type), samples with *TP53* mutations once again exhibited a statistically significant increase in average number of FM panel amplifications compared to *TP53* wild-type samples (3.3 FM panel amplifications vs. 1.8 FM panel amplifications, p < .001).

**Table 1.** Amplificators^1,2^ versus non-amplificators in both the TCGA and UCSD datasets.

Information for TCGA and for UCSD Cancer Cohort									
		Total samples N (% total)		*TP53*-mutated samples N (% total)		Number of WGS amplifications per sample, mean [CI_95%_]		Number of FM-panel amplifications per sample, mean [CI_95%_]	
TCGA Data*									
All samples		7246 (100)		2897 (40)		344 [331–357]		5.1 [4.9–5.3]	
Non-amplificators (WGS)		6519 (100)		2448 (38)		190 [184–197]		3.2 [3.1–3.3]	
WGS amplificators^1^		727 (100)		449 (62)		1720 [1662–1778]		22.4 [21.5–23.2]	
Non-amplificators (FM)		6514 (100)		2416 (37)		207 [199–215]		3.0 [2.9–3.1]	
FM-panel amplificators^2^		732 (100)		481 (66)		1562 [1503–1621]		24.0 [23.2–24.7]	
UCSD**									
All samples		1891 (100)		824 (44)		-		2.5 [2.2–2.7]	
Non-amplificators (FM)		1706 (100)		707 (41)		-		1.2 [1.1–1.3]	
FM-panel amplificators^2^		185 (100)		117 (63)		-		14.2 [13.4–15.0]	
Amplifications in *TP53* mutated vs. wild-type samples in the TCGA and UCSD datasets									
	Total samples N (% total)		Number of WGS amplifications per sample^1^ Mean [CI_95%_] Median [range]		p-value		Number of FM panel amplifications per sample^2^ Mean [CI_95%_] Median [range]		p-value
TCGA Data*									
*TP53* mutated samples	2897 (40%)		516 [491–542] 289 [0–9658]		6.58E-83		7.7 [7.3–8.0] 5 [0–93]		9.28E-96
*TP53* wild-type samples	4349 (60%)		229 [215–243] 7 [0–6981]				3.4 [3.2–3.6] 0 [0–73]		
UCSD**									
*TP53* mutated samples	824 (44%)		-		-		3.3 [3.0–3.7] 1 [0–32]		3.39E-13
*TP53* wild-type samples	1067 (56%)		-				1.8 [1.5–2.0] 0 [0–38]		

*In the TCGA data, a total of 7,246 samples that had copy number variation (CNV) and mutation data were curated from 11,245 possible TCGA samples across all cancer cohorts.

^1^The phenotype “WGS amplificator” corresponds to tumors presenting a high number of amplifications considering the whole genome (top 10% amplification burden, within the whole genome): All p < 0.0001.

^2^The phenotype “FM-panel amplificator” corresponds to tumors presenting a high number of amplifications considering only genes included in the Foundation One panel (top 10% amplification burden, within the 315 genes of the Foundation One panel manufactured by Foundation Medicine). All p < 0.0001.

**In the UCSD data, a total of 1,891 samples sequenced from patients treated at Moores Cancer Center in La Jolla, CA, were analyzed across all cancer types.

**Abbreviations**: CI_95%_ = 95% confidence interval; FM = Foundation Medicine; N = number; TCGA = The Cancer Genome Atlas; UCSD = University of California San Diego; WGS = whole genome sequencing.

Additional analysis compared the frequency of mutated genes in samples with amplification burden in the top 10% of samples vs. those in the bottom 90% using WGS or the FM panel (Table 2). There was a significant association in the TCGA dataset between alterations in *TP53, BRAF*, and *KRAS* and tumor amplification burden in both the FM panel as well as WGS. *TP53* alterations were associated with increased amplifications, while *BRAF* and *KRAS* alterations associated with decreased amplifications (all p < .01). In the UCSD dataset analyzed with the FM panel, only *TP53* alterations (but not *BRAF* or *KRAS* alterations) were found to be significantly associated with increased tumor amplification burden (perhaps because this dataset was smaller and WGS was not available) (all p < .01 for significance).

**Table 2.** Comparing frequency of mutated genes in samples with amplification burden in the top 10% of samples vs. those in the bottom 90% using WGS or the FM panel (see also Supplemental Table S1 for the FM gene list) for both TCGA and UCSD datasets.

Gene	Top 10% of amplifications	Bottom 90% of amplifications			Odds Ratio [CI_95%_]	Bonferroni adjusted p-value	
TCGA dataset comparing frequency of mutated genes in samples with amplifications in the top 10% vs. those in the bottom 90% using WGS*							
TP53****	**449/727 (61.8%)**		**2448/6519 (37.6%)**	**2.7 [2.3–3.1]**		**<0.00001**	*TP53* alterations associated with increased amplifications while *BRAF* and *KRAS* alterations associated with decreased amplifications
*BRAF*	17/727 (2.3%)		591/6519 (9.1%)	0.2 [0.1–0.4]		**<0.00001**	
*KRAS*	34/727 (4.7%)		611/6519 (9.4%)	0.5 [0.3–0.7]		**0.008**	
*GATA3*	29/727 (4.0%)		159/6519 (2.4%)	1.7 [1.1–2.5]		Not significant	
TCGA dataset comparing frequency of mutated genes in samples with amplifications in the top 10% vs. those in the bottom 90% using FM panel**							
*TP53*	**481/732 (65.7%)**		**2416/6514 (37.1%)**	**3.3 [2.8–3.8]**		**<0.003**	*TP53* alterations associated with increased amplifications while *BRAF* and *KRAS* alterations associated with decreased amplifications
*BRAF*	16/732 (2.2%)		592/6514 (9.1%)	0.2 [0.1–0.4]		**<0.003**	
*KRAS*	25/732 (3.4%)		620/6514 (9.5%)	0.3 [0.2–0.5]		**<0.003**	
*GATA3*	32/732 (4.4%)		156/6514 (2.4%)	1.9 [1.3–2.7]		Not significant	
UCSD dataset comparing frequency of mutated genes in samples with amplifications in the top 10% vs. those in the bottom 90% using FM panel***							
*TP53*	**117/185 (63.2%)**	**707/1706 (41.4%)**			**2.4 [1.8–3.3]**	**<0.003**	*TP53* alterations associated with increased amplifications
*BRAF*	4/185 (2.2%)	107/1706 (6.3%)			0.3 [0.1–0.9]	Not significant	
*KRAS*	32/185 (17.3%)	259/1706 (15.2%)			1.2 [0.8–1.7]	Not significant	
*GATA3*	11/185 (5.9%)	35/1706 (2.1%)			3.0 [1.5–6.0]	Not significant	

*Using the TCGA cohort, which consists of 7,246 cancer samples, the 90^th^ percentile for number of amplifications using WGS was calculated to be 1,016. The 90^th^ percentile for number of amplifications using the FM panel was calculated to be 15.

** The FM panel includes 321 genes (see supplemental Table S1).

*** Using the UCSD dataset, which consists of 1,891 cancer samples, the 90^th^ percentile for number of amplifications (FM panel) was calculated to be 9.

**** Refers to number of samples with designated gene mutation/total samples in that subgroup. For instance, within TCGA database, 727 samples were in the top 10% for amplification burden (“amplificators”); of these 727 samples, 449 (65.7%) had a *TP53* mutation.

**Abbreviations**: FM = Foundation Medicine; TCGA = The Cancer Genome Atlas; UCSD = University of California San Diego; WGS = whole genome sequencing.

## Conclusions

The tumor suppressor *TP53* has long been implicated in the development of diverse cancers. It is the most commonly mutated gene in cancer and has diverse functions important to oncogenesis.^12–17^ Unfortunately, *TP53* alterations are considered difficult to target from a therapeutic standpoint.^12–17^ Groups have, however, reported an increase in the expression of vascular endothelial growth factor (VEGF) in a pan-cancer analysis as well as improvement in outcome of patients who receive VEGF/VEGFR inhibitors when their tumors harbor deleterious *TP53* alterations as a possible therapeutic proxy for targeting harmful *TP53* alterations.^13,15,17^

Importantly, mutations in the *TP53* tumor suppressor increase genomic instability, corroborating the reputation of *TP53* as the ”guardian of the genome.”^12^ Interestingly, TP53 also likely plays a role in transcriptional regulation.^18^ This inherent process of upregulating and downregulating various aspect of mRNA production may directly impact the tumor suppression function of this protein.^18,19^ It may be that TP53 impacts mRNA expression through many TP53-dependent pathways that directly impact transcriptional regulation and via indirect transcriptional regulation (such as by virtue of secondary amplifications) as well as through posttranscriptional regulation.^18^

Our data suggest that a subset of cancers have high tumor amplification burden, and these tumors are significantly more likely to bear *TP53* mutations than those with lower tumor amplification burden. In contrast, *BRAF* and *KRAS* alterations correlated with decreased tumor amplification burden in the TCGA dataset. A limitation of our findings is that it is unclear why *BRAF* and *KRAS* alterations would correlate with a decreased tumor amplification burden. It is also unclear why specific tumor types such as breast cancer and ovarian serous carcinomas are especially likely to have an amplificator phenotype, though the latter could be due to the fact that high-grade ovarian serous carcinomas demonstrate *TP53* anomalies in about 90% of cases.^20^ *TP53* mutations may correlate with high tumor amplification burden because these mutations impair genomic stability as evidenced by loss-of-function *TP53* mutations shown to be associated with increased mutation rate.^21^ Since amplifications (which generally [but not always] cause overexpression)^22^ may be pharmacologically tractable in some cases, targeting them may be an indirect way to impact the consequences of *TP53* mutation-related genomic instability.

## Methods

Two distinct datasets were used for the statistical analysis. The first dataset was retrieved from the publicly available repository, The Cancer Genome Atlas (TCGA) (https://portal.gdc.cancer.gov/), which is a cohort of sequenced cancer samples from patients. Our second dataset was composed of a cohort of patients who had been treated and sequenced using the FoundationOne CDx gene panel (Foundation Medicine, Inc., Cambridge, MA) (https://corpsite.foundationmedicine.com/genomic-testing) (Supplemental Table S1) at UCSD. All studies were conducted under the auspices of an Internal Review Board (IRB) Committee-approved protocol (NCT02478931) and any investigational trials for which the patient gave consent.

Data collected and reviewed retrospectively for this study from the UCSD cohort included genomic information from sequencing results detailing amplifications and mutations present (with gene localization) across all cancer types and using the genes in the FM panel. Data collected from TCGA included demographic information such as age (years), sex, primary cancer diagnosis, number of amplifications, and mutation status for all of the FM panel genes. Within the TCGA dataset, patient samples from all cancer types were queried.

Descriptive statistics were tabulated to describe patient sample information, comparing our amplificators to non-amplificators in both the TCGA cohort, as well as our institutional dataset. For the TCGA cohort, statistical summaries were stratified into sub-groups based on amplificator vs. non-amplificator phenotype, as determined by both WGS and the FM gene panels (Supplemental Tables S2 and S3). Descriptive information included number of patients matching the criteria for amplificator or non-amplificator phenotype, total number of *TP53* mutated samples present in each sub-group, average number of WGS amplifications per sample, and average number FM gene amplifications per sample. Similar analysis was conducted in the UCSD cohort; however, amplificators were determined solely based on number of amplifications present in the FM gene panel as WGS was not utilized for these patients. Additionally, for both cohorts, a second analysis was conducted to describe summary statistics based on *TP53* mutation status. Patient samples were stratified into *TP53* mutant and *TP53* wild-type sub-groups. Within these two sub-groups, the average number of WGS amplifications and FM-panel amplifications per sample was determined and compared using student’s *t*-tests. Similar analysis was conducted for the UCSD dataset comparing only number of FM panel amplifications per sample in the *TP53* mutant and wild-type sub-groups.

Further analysis was performed to assess the number of mutations in common cancer genes (Supplemental Table S1) across samples with the top 10% of amplifications against samples with the bottom 90% of amplifications. The top four genes reported in our analysis were *TP53, BRAF, KRAS, and GATA3*. Odds ratios and Bonferroni adjusted p-values were then calculated to compare mutation burden between the two amplification sub-groups. This analysis was performed using both the WGS and FM information for the TCGA dataset and only FM-panel genes for the UCSD dataset.

For the TCGA dataset, copy numbers were measured using whole-genome microarray. Gene-level focal copy number variations (CNVs) were normalized using data from all TCGA cohorts (« pan-cancer » data set) and estimated using the GISTIC2 threshold method,^23^ where the values −2, −1, 0, 1, and 2 represented homozygous deletion, single-copy deletion, diploid normal copy, low-level amplification, and high-level amplification, respectively. Only high-level amplification (+2) was considered for this analysis.

For the UCSD dataset, copy numbers were measured using gene-panel capture sequencing (FoundationOne CDx test, Foundation Medicine, Inc.) and gene-level amplifications were reported when the number of copies exceeded 6.

All statistical analysis was conducted using a combination of Microsoft Excel version 16.42 (Microsoft Corporation, Redmond, Washington, USA) and R version 3.6.1 (R Foundation for Statistical Computing, Vienna, Austria).

## Supplementary Material

Supplemental Material

## Data Availability

The datasets used and/or analyzed during the current study are available from the corresponding author on reasonable request.
