# Supplementary material for: High tumor amplification burden is associated with TP53 mutations in the pan-cancer setting
Source: Cancer Biol Ther. 2022 Sep 28;23(1):1–6. doi: 10.1080/15384047.2022.2128608 (PMC9542347; doi:10.1080/15384047.2022.2128608)
Supplement: Supplemental Material [file KCBT_A_2128608_SM2356.docx]

**Supplemental Table 1: Complete list of genes found in the FoundationOne CDx panel, a sequencing panel that**

**highlights commonly associated oncological genes.**

**Supplemental Table 2: Demographic table comparing patient characteristics in the TCGA cohort between WGS amplificators^1^ versus non-amplificators.**

|  | **Non-amplificators**  **N=6519 (90%)** | **WGS amplificators**  **N=727 (10%)** | **p-value^2^** | **Bonferroni-adjusted**  **p-value^3^** |
| --- | --- | --- | --- | --- |
| **Age, Mean (SD in years)** | 58.84 (14.67) | 60.62 (13.59) | **0.002** | **0.002** |
| **Sex, N (% non/WGS amplificators)** |  |  |  |  |
| Female | 3168 (87.2) | 467 (12.8) | **< 0.0001** | **< 0.0001** |
| Male | 2899 (93.1) | 215 (6.9) | **< 0.0001** | **< 0.0001** |
| Unknown | 452 (90.9) | 45 (9.1) | *0.486* | *n.s.* |
| **Diagnosis, N (% non/WGS amplificators)** |  |  |  |  |
| Breast Invasive Carcinoma | 673 (10.3) | 180 (24.8) | **< 0.0001** | **< 0.0001** |
| Ovarian Serous Carcinoma | 256 (3.9) | 178 (24.5) | **< 0.0001** | **< 0.0001** |
| Lung Adenocarcinoma | 406 (6.2) | 62 (8.5) | **0.021** | *n.s.* |
| Bladder Urothelial Carcinoma | 339 (5.2) | 47 (6.5) | *0.163* | *n.s.* |
| Lung Squamous Cell Carcinoma | 144 (2.2) | 32 (4.4) | **0.0008** | **0.024** |
| Head and Neck Squamous Cell Carcinoma | 454 (7.0) | 31 (4.3) | **0.005** | *n.s.* |
| Sarcoma | 158 (2.4) | 28 (3.9) | **0.026** | *n.s.* |
| Endometrial Carcinoma | 213 (3.3) | 28 (3.9) | *0.384* | *n.s.* |
| Hepatocellular Carcinoma | 157 (2.4) | 24 (3.3) | *0.166* | *n.s.* |
| Stomach Adenocarcinoma | 255 (3.9) | 20 (2.8) | *0.126* | *n.s.* |
| Cutaneous Melanoma | 264 (4.0) | 19 (2.6) | *0.068* | *n.s.* |
| Uterine Carcinosarcoma | 42 (0.6) | 13 (1.8) | **0.003** | *n.s.* |
| Lower Grade Glioma | 491 (7.5) | 11 (1.5) | **< 0.0001** | **< 0.0001** |
| Prostate Adenocarcinoma | 245 (3.8) | 9 (1.2) | **0.0002** | **0.005** |
| Pancreatic Adenocarcinoma | 93 (1.4) | 6 (0.8) | *0.237* | *n.s.* |
| Colon Adenocarcinoma | 357 (5.5) | 5 (0.7) | **< 0.0001** | **< 0.0001** |
| Cervical Squamous Cell/Endocervical Carcinoma | 163 (2.5) | 4 (0.6) | **0.0002** | **0.006** |
| Adrenocortical Carcinoma | 46 (0.7) | 4 (0.6) | *0.814* | *n.s.* |
| Pheochromocytoma / Paraganglioma | 74 (1.1) | 4 (0.6) | *0.184* | *n.s.* |
| Kidney Clear Cell Carcinoma | 388 (6.0) | 3 (0.4) | **< 0.0001** | **< 0.0001** |
| Thyroid Carcinoma | 323 (5.0) | 3 (0.4) | **< 0.0001** | **< 0.0001** |
| Glioblastoma Multiforme | 255 (3.9) | 3 (0.4) | **< 0.0001** | **< 0.0001** |
| Cholangiocarcinoma | 31 (0.5) | 3 (0.4) | *1.000* | *n.s.* |
| Uveal Melanoma | 74 (1.1) | 3 (0.4) | *0.084* | *n.s.* |
| Testicular Germ Cell Tumors | 120 (1.8) | 2 (0.3) | **0.0004** | **0.012** |
| Rectum Adenocarcinoma | 117 (1.8) | 2 (0.3) | **0.0006** | **0.017** |
| Diffuse Large B-cell Lymphoma | 39 (0.6) | 2 (0.3) | *0.430* | *n.s.* |
| Acute Myeloid Leukemia | 156 (2.4) | 1 (0.1) | **< 0.0001** | **< 0.0001** |
| Kidney Papillary Cell Carcinoma | 129 (2.0) | 0 (0.0) | **< 0.0001** | **< 0.0001** |
| Kidney Chromophobe | 57 (0.9) | 0 (0.0) | **0.004** | *n.s.* |

^1^ WGS amplificators corresponds to tumors presenting a high number of amplifications considering the whole genome (top 10% amplification burden). **Breast invasive and ovarian serous carcinomas are more likely to present a WGS-amplificator phenotype.**

^2^ P-values were obtained for each variable comparing to the remaining of the cohort. P-values <0.05 were considered significant.

^3^ Bonferroni-adjusted p-values were obtained by multiplying the initial p-value by the number of comparisons made, respectively p-value/3 for the sex-related variables and p-value/30 for the diagnosis-related variables. Adjusted p-values <0.05 were considered significant.

Abbreviations: N=number; n.s.=non-significant; SD=standard deviation; WGS=whole-genome sequencing.**Supplemental Table 3: Demographic table comparing patient characteristics in the TCGA cohort between FM**-**panel amplificators^1^ versus non-amplificators.**

|  | **Non-amplificators**  **N=6514 (90%)** | **FM**-**panel amplificators**  **N=732 (10%)** | **p-value^2^** | **Bonferroni-adjusted**  **p-value^3^** |
| --- | --- | --- | --- | --- |
| **Age, Mean (SD in years)** | 58.73 (14.70) | 61.58 (13.11) | **<0.001** | **<0.001** |
| **Sex, N (% non/FM-panel amplificators)** |  |  |  |  |
| Female | 3175 (87.3) | 460 (12.7) | **< 0.0001** | **< 0.0001** |
| Male | 2891 (92.8) | 223 (7.2) | **< 0.0001** | **< 0.0001** |
| Unknown | 448 (90.1) | 49 (9.9) | *0.939* | *n.s.* |
| **Diagnosis, N (% non/FM-panel amplificators)** |  |  |  |  |
| Ovarian Serous Carcinoma | 258 (4.0) | 176 (24.0) | **<0.0001** | **<0.0001** |
| Breast Invasive Carcinoma | 692 (10.6) | 161 (22.0) | **<0.0001** | **<0.0001** |
| Lung Adenocarcinoma | 411 (6.3) | 57 (7.8) | *0.131* | *n.s.* |
| Bladder Urothelial Carcinoma | 335 (5.1) | 51 (7.0) | **0.045** | *n.s.* |
| Head and Neck Squamous Cell Carcinoma | 435 (6.7) | 50 (6.8) | *0.876* | *n.s.* |
| Lung Squamous Cell Carcinoma | 136 (2.1) | 40 (5.5) | **<0.0001** | **<0.0001** |
| Uterine Corpus Endometrial Carcinoma | 209 (3.2) | 32 (4.4) | *0.102* | *n.s.* |
| Sarcoma | 155 (2.4) | 31 (4.2) | **0.004** | *n.s.* |
| Stomach Adenocarcinoma | 247 (3.8) | 28 (3.8) | *0.919* | *n.s.* |
| Hepatocellular Carcinoma | 159 (2.4) | 22 (3.0) | *0.380* | *n.s.* |
| Uterine Carcinosarcoma | 38 (0.6) | 17 (2.3) | **<0.0001** | **0.0005** |
| Cutaneous Melanoma | 268 (4.1) | 15 (2.0) | **0.005** | *n.s.* |
| Lower Grade Glioma | 493 (7.6) | 9 (1.2) | **<0.0001** | **<0.0001** |
| Cervical Squamous Cell / Endocervical Carcinoma | 160 (2.5) | 7 (1.0) | **0.009** | *n.s.* |
| Kidney Clear Cell Carcinoma | 385 (5.9) | 6 (0.8) | **<0.0001** | **<0.0001** |
| Prostate Adenocarcinoma | 248 (3.8) | 6 (0.8) | **<0.0001** | **<0.0001** |
| Pancreatic Adenocarcinoma | 94 (1.4) | 5 (0.7) | *0.127* | *n.s.* |
| Cholangiocarcinoma | 30 (0.5) | 4 (0.5) | *0.772* | *n.s.* |
| Glioblastoma Multiforme | 255 (3.9) | 3 (0.4) | **<0.0001** | **<0.0001** |
| Adrenocortical Carcinoma | 47 (0.7) | 3 (0.4) | *0.479* | *n.s.* |
| Colon Adenocarcinoma | 360 (5.5) | 2 (0.3) | **<0.0001** | **<0.0001** |
| Uveal Melanoma | 75 (1.2) | 2 (0.3) | **0.022** | *n.s.* |
| Thyroid Carcinoma | 325 (5.0) | 1 (0.1) | **<0.0001** | **<0.0001** |
| Acute Myeloid Leukemia | 156 (2.4) | 1 (0.1) | **<0.0001** | **<0.0001** |
| Testicular Germ Cell Tumors | 121 (1.9) | 1 (0.1) | **<0.0001** | **0.002** |
| Rectum Adenocarcinoma | 118 (1.8) | 1 (0.1) | **<0.0001** | **0.002** |
| Diffuse Large B-cell Lymphoma | 40 (0.6) | 1 (0.1) | *0.120* | *n.s.* |
| Kidney Papillary Cell Carcinoma | 129 (2.0) | 0 (0.0) | **<0.0001** | **<0.0001** |
| Pheochromocytoma / Paraganglioma | 78 (1.2) | 0 (0.0) | **0.0004** | **0.013** |
| Kidney Chromophobe | 57 (0.9) | 0 (0.0) | **0.004** | *n.s.* |

^1^ FM-panel amplificators corresponds to tumors presenting a high number of amplifications considering only genes included in the Foundation One panel (top 10% amplification burden). **Breast invasive and ovarian serous carcinomas are more likely to present a FM-amplificator phenotype.**

^2^ P-values were obtained for each variable comparing to the remaining of the cohort. P-values <0.05 were considered significant.

^3^ Bonferroni-adjusted p-values were obtained by multiplying the initial p-value by the number of comparisons made, respectively p-value/3 for the sex-related variables and p-value/30 for the diagnosis-related variables. Adjusted p-values <0.05 were considered significant.

Abbreviations: N=number; n.s.=non-significant; SD=standard deviation; FM=Foundation Medicine.
